# Articulated Structures of D-A Type Dipolar Dye with AIEgen: Synthesis, Photophysical Properties, and Applications

**DOI:** 10.3390/ma13081939

**Published:** 2020-04-20

**Authors:** Na Hee Kim, Byeong Wook Kim, Youngseo Kim, Junho K. Hur, Junyang Jung, Yohan Oh, Sungnam Park, B. Moon Kim, Dokyoung Kim

**Affiliations:** 1 Department of Biomedical Science, Graduate School, Kyung Hee University, Seoul 02447, Korea; pionaheek@gmail.com (N.H.K.); jhur@khu.ac.kr (J.K.H.); jjung@khu.ac.kr (J.J.); 2 Department of Chemistry, College of Natural Sciences, Seoul National University, Seoul 08826, Korea; kbo0528@snu.ac.kr; 3 Department of Chemistry, Korea University, Seoul 02841, Korea; youngseo1110@hanmail.net; 4 Department of Pathology, College of Medicine, Kyung Hee University, Seoul 02447, Korea; 5 Department of Anatomy and Neurobiology, College of Medicine, Kyung Hee University, Seoul 02447, Korea; 6 Department of Biomedical Science, Graduate School of Biomedical Science and Engineering, Hanyang University, Seoul 04763, Korea; 7 Department of Biochemistry and Molecular Biology, College of Medicine, Hanyang University, Seoul 04763, Korea; 8 Center for Converging Humanities, Korea University, Seoul 02841, Korea; 9 Medical Research Center for Bioreaction to Reactive Oxygen Species and Biomedical Science Institute, School of Medicine, Graduate School, Kyung Hee University, Seoul 02841, Korea

**Keywords:** fluorophore, dipolar dye, functionalized naphthalene, aggregation-induced emission, DMSO sensing

## Abstract

Articulated structures of naphthalene-based donor (D)-acceptor (A) type dipolar dye and aggregation-induced emission luminogen (AIEgen) based on tetraphenylethylene (TPE) were synthesized, and their photophysical properties were analyzed for the first time. There are many fluorophore backbones, which have dipolar structure and AIEgen. However, there has been neither property analysis nor research that closely articulates DA and AIE through non-conjugation linker. We have therefore prepared two representative fluorophores; DA-AIE series (DA-AIE-M and DA-AIE-D), and characterized their UV/vis absorption and emission properties with quantum chemical calculations. In addition, we utilized the unique photophysical properties of DA-AIE-D for monitoring a trace of dimethyl sulfoxide (DMSO) in aqueous media, including real water samples.

## 1. Introduction

Donor-bridge-acceptor (D-π-A, D-A) type dipolar fluorophores have been widely used as molecular probes and biological tags due to their unique photophysical properties, such as high quantum yield, environment sensitive emission, high biocompatibility with photostability, and multi-photon absorption capability [1,2,3,4,5,6]. To date, many D-A type dipolar dyes have been introduced, including 6-acetyl-2-(dimethylamino)-naphthalene (named, Acedan), 4-amino (or hydroxy)-1,8-naphthalimide, 7-amino (or hydroxy)-4-nitro-benzoxadiazole (NBD), coumarin, and cyanine (Cy) [7,8,9,10,11,12,13]. The photophysical properties of these fluorophores could be readily tunable by changing (i) the functional group at donor and acceptor site, (ii) the size of the bridge linker, and (iii) the physical property; aggregation/disaggregation.

Typically, D-A type fluorophores emit fluorescence in aqueous solution and their emission properties depend significantly on the solvent polarity, due to the intramolecular charge-transfer (ICT) character in the excited state [1,14]. In an aggregated form of D-A type fluorophore (solid state), it generally shows no emission, although the emission characteristics do change depending on the molecular arrangements in the aggregated form [15].

Unlike the D-A type fluorophores, AIEgens show strong emissions in an aggregated form (solid state), but no emission is observed in a dissolved form (solution state) [11]. This unique property of AIEgen has been applied in various fields, basic research as well as industries [16]. Recently, it showcased in a few cases that the photophysical properties of AIEgens are manageable, like D-A type fluorophores, by introducing electron donating and accepting moieties to the AIEgen backbones through π-conjugation [17,18,19]. However, the synthesis, photophysical properties, and practical applications of the articulated structures (no π-conjugation), between D-A type fluorophore and AIEgen have yet to be fully investigated.

**Figure 1:**
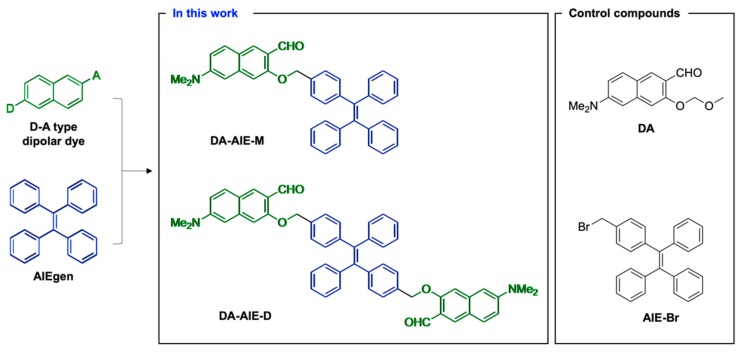
Chemical structure of D-A type naphthalene-based dipolar dye and tetraphenylethylene-based AIEgen. In this work: articulated structures of D-A type dipolar dye with AIEgen; DA-AIE-M and DA-AIE-D. Control compounds: non-articulated compounds on behalf of dipolar dye (DA) and AIEgen (AIE-Br).

Herein, we have comprehensively studied two compounds; the DA-AIE series (DA-AIE-M, DA-AIE-D), which have articulated structures of 6-(dimethylamino)-2-naphthaldehyde (DA) and tetraphenyl-ethylene (TPE), as a representative D-A type fluorophore and AIEgen, respectively (Figure 1). The DA was linked to TPE via a C-C bond in a non-conjugated manner; DA-AIE-M: mono-DA-substituted TPE, DA-AIE-D: bis-DA-substituted TPE. In this study, the photophysical properties of these two compounds were experimentally characterized within various environments and in conjunction with quantum chemical calculations. Its unique properties were demonstrated for the detection of residual DMSO in real water samples with high sensitivity.

## 2. Materials and Methods

The chemical reagents were purchased from Aldrich (St. Louis, MO, USA), TCI (Tokyo, Japan), Alfa Aesar (Ward Hill, MA, USA), and Acros Organics (Morris Plains, NJ, USA). Species used to perform the screening of metal ions, and amino acid, and hydrazine; AuCl_3_, FeCl_2_, FeCl_3_, CaCl_2_, CuCl_2_, HgCl_2_, (C_2_H_5_)_3_PAuCl, CdCl_2_, NiCl_2_, NaCl_2_, PdCl_2_, KCl, L-Cysteine, L-Glutathione, L-Lysine, DL-Homocysteine, and N_2_H_4_. Commercially available reagents and anhydrous solvents were used without further purification. Chemical reactions were performed under an argon atmosphere. Coumarin 153 and 9,10-diphenylanthracene (DPA) were used for the quantum yield measurement. Thin-layer chromatography (TLC) was performed on the pre-coated silica gel 60F-254 glass plates (Merck KgaA, Darmstadt, Germany). ^1^H NMR spectra were recorded on an Agilent 400-MR DD2 (400 MHz) and ^13^C NMR spectra were recorded on a Varian/Oxford As-500 (125 MHz) in the indicated solvent. In the NMR spectra, the chemical shifts (δ) are reported in parts per million (ppm), relative to the signal (0.00 ppm), with an internal standard tetramethylsilane (TMS) for the solution in DMSO-*d6* (2.50 ppm for ^1^H and 39.52 ppm for ^13^C) or CDCl_3_ (7.26 ppm for ^1^H and 77.16 ppm for ^13^C). Multiplicities are indicated by s (singlet), d (doublet), dd (doublet of doublet), and m (multiplet). Coupling constants were reported in Hz. High resolution mass spectrometry (HRMS) analysis of the compounds were conducted using a Ultra High Resolution ESI Q-TOF mass spectrometer (Bruker, Billerica, MA, USA), from the Organic Chemistry Research Center at Sogang University, Seoul, Korea.

### 2.1. Synthesis

**Figure 2:**
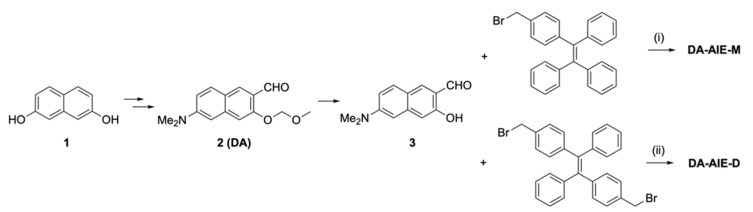
Synthetic scheme for DA-AIE-M and DA-AIE-D. Reagents and conditions: (**i**) NaH, DMF, 25 °C, 1 h, 58%; (**ii**) NaH, DMF, 25 °C, 1 h, 30%.

The procedures used in the synthesis of DA-AIE series are summarized in Figure 2. Compound 3 was prepared by following the existing methods (4 step synthesis from naphthalene-2,7-diol) [20].

*6-(dimethylamino)-3-((4-(1,2,2-triphenylvinyl)benzyl)oxy)-2-naphthaldehyde (DA-AIE-M)*. NaH (13.9 mg, 0.348 mmol) and compound 3 (50.0 mg, 0.232 mmol) were dissolved in *N*,*N*-dimethylformamide (DMF, 0.77 mL). The reaction mixture was stirred for 30 min at 0 °C. Then, (2-(4-(bromomethyl)phenyl)ethene-1,1,2-triyl)-tribenzene (108.6 mg, 0.255 mmol) was added, and the mixture was stirred for an additional 1 h at 25 °C. The crude reaction mixture was quenched by adding deionized H_2_O (DI H_2_O) and diluted with ethyl acetate (EtOAc). The organic layer was washed with DI H_2_O, dried over anhydrous magnesium sulfate (MgSO_4_), and concentrated in vacuo. The residue was purified by flash column chromatography (*n*-hex/EtOAc = 9:1, *v/v*) to produce the DA-AIE-M (75.2 mg, 58%, yellowish green solid). ^1^H NMR (400 MHz, DMSO-*d*_6_): δ 3.07 (s, 6H), 5.20 (s, 2H), 6.80 (d, J = 2.5 Hz, 1H), 6.96–7.05 (m, 8H), 7.09–7.14 (m, 10H), 7.22 (s, 1H), 7.32 (J = 8.0 Hz, 2H), 7.82 (d, J = 9.1 Hz, 1H), 8.15 (s, 1H), 10.32 (s, 1H); ^13^C NMR (500 MHz, CDCl_3_): δ 40.4, 70.2, 104.2, 105.5, 114.5, 121.0, 122.4, 126.6, 126.6, 126.6, 126.7, 127.8, 130.8, 130.8, 131.4, 131.4, 131.4, 131.7, 134.7, 139.9, 140.6, 141.5, 143.7, 143.7, 143.7, 143.8, 150.9, 157.7, 189.7. HRMS (*m/z*): [M + Na]^+^ calcd for C_40_H_33_NNaO_2_, 582.2404; found, 582.2404.

*(E)-3,3′-((((1,2-diphenylethene-1,2-diyl)bis(4,1-phenylene))bis(methylene))bis(oxy))bis(6-(dimethylamino)-2-naphthaldehyde) (DA-AIE-D)*. NaH (9.90 mg, 0.251 mmol) and compound 3 (43.6 mg, 0.203 mmol) were dissolved in DMF (0.6 mL). The reaction mixture was stirred for 30 min at 0 °C. Then, (*E*)-1,2-bis(4-(bromomethyl)phenyl)-1,2-diphenylethene (50.0 mg, 0.0965 mmol) was added, and stirred for another 1 h at 25 °C. The crude mixture was quenched by adding DI H_2_O and diluted with EtOAc. The organic layer was washed with DI H_2_O, dried over anhydrous MgSO_4_, and concentrated in vacuo. The residue was purified by flash column chromatography (*n*-hex/EtOAc = 7:3, *v*/*v*) to produce the DA-AIE-D (22.5 mg, 30%, yellowish green solid). ^1^H NMR (400 MHz, DMSO-*d*_6_): δ 3.05 (d, J = 8.2 Hz, 12H), 5.17 (d, J = 12.0 Hz, 4H), 6.80 (s, 2H), 6.97-7.15 (m, 16H), 7.21 (s, 2H), 7.31 (d, J = 7.9 Hz, 4H), 7.81 (dd, J = 6.1, 9.2 Hz, 2H), 8.14 (d, J = 3.5 Hz, 2H), 10.31 (d, J = 3.2 Hz, 2H); ^13^C NMR (500 MHz, CDCl_3_): δ 40.5, 70.2, 104.2, 105.5, 105.6, 114.6, 113.0, 122.4, 126.8, 126.8, 127.9, 130.8, 130.9, 131.4, 131.5, 131.7, 134.8, 140.0, 141.0, 143.6, 150.9, 157.8, 189.7. HRMS (*m*/*z*): [M + Na]^+^ calcd for C_54_H_46_N_2_NaO_4_, 809.3350; found, 809.3348.

### 2.2. UV/Vis Absorption and Fluorescence Assay

UV/Vis absorption spectra were obtained using a spectrophotometer (Agilent Technologies Cary 8454, Santa Clara, CA, USA). Fluorescence emission spectra were recorded on a spectro-fluorophotometer (SHIMADZU CORP. RF-6000, Tyoto, Japan), with a 1-cm standard quartz cell (internal volume of 1 mL, 108-000-10-40 (10 mm), 108-F-10-40 (10 × 4 mm); Hellma Analytics, Müllheim, Germany). The stock solution of each compound (10 mM) was prepared by dissolving it in DMSO. Absorption and emission spectra were recorded with 10 μM of each compound, in various solvents at 25 °C. To measure the emission spectrum, the sample solution was excited at the maximum absorption wavelength. Limit of detection (LOD) was calculated based on a signal-to-noise (S/N) criteria ratio of more than 3 from the concentration-dependent emission spectra.

### 2.3. Quantum Chemical Calculations

Quantum chemical calculations were carried out using the density functional theory (DFT) and time-dependent DFT methods with the B3LYP-D3 functional and 6-31G(d) basis set that is implemented in the Gaussian 16 package [21]. The optimized structures, electronic energies, frontier orbitals (HOMO and LUMO), natural transition orbitals (NTOs), electronic absorption spectrum, and emission spectrum were calculated. We used the integral equation formalism polarizable continuum (IEF-PCM) model for solvation.

### 2.4. Sensing Applications in Real Water Samples

Real water samples were collected from (i) Tap water (Kyung Hee University, Seoul, Korea), (ii) Commercial bottled drinking water (Jeju Samdasoo, Korea), (iii) Purified drinking water (Kyung Hee University, Seoul, Korea), (iv) Sea water (Oido, Gyeonggi-do, Korea), (v) River water (Han-river, Seoul, Korea), and (vi) Lake water (Jemyoung lake, Seoul, Korea). The real water samples were spiked with DMSO and used for the analysis.

## 3. Results and Discussion

### 3.1. Material Design, Synthesis, and Characterization

First, we chose 6-(dimethylamino)-3-hydroxy-2-naphthaldehyde (compound 3) as a representative D-A type fluorophore, because its hydroxy moiety could be linked with AIEgen material without disturbing the character of dipolar backbone. The synthesis and distinguishable applications of compound 3 have been presented by our research group [22,23,24,25,26,27]. For an AIEgen material, we chose bromomethyl-functionalized TPE because it showed a typical property of AIEgen and has been widely used throughout various research fields [28,29,30]. D-A type fluorophore and AIEgen have shown to exhibit opposite photophysical properties. Compound 3 and TPE were directly linked via sp^3^ carbon linker (-CH_2_-) to give DA-AIE-M (mono-DA-conjugated), DA-AIE-D (bis-DA-conjugated) (Figure 1). This is the first time the articulation of dipolar D-A type fluorophore and AIEgen in a single molecular structure was reported, which shows significantly different photophysical properties from individual D-A type fluorophore and AIEgen. In this study, we tried to identify the photophysical properties of DA-AIE-M and DA-AIE-D and understand the dominant factors that influence their emission properties; either the environment-sensitive properties from dipolar dye or the AIE properties from TPE. We measured UV/vis absorption and emission spectra of the compounds dissolved in various solvents and carried out quantum chemical calculations to estimate the optimized structures and optical properties of DA-AIE-M and DA-AIE-D.

The D-A type dipolar fluorophore (compound 3) was prepared from 2,7-dihydroxy-naphthalene (compound 1) via four-step synthesis (Figure 2) [20]; (i) Bucherer reaction, (ii) methoxy-methyl ether (MOM) protection, (iii) directed lithiation using *t*-BuLi (resulting product: compound 2), and (iv) MOM deprotection in acidic solution. Then, the DA-AIE series were produced through the reaction between compound 3 and bromomethyl-TPE in the presence of sodium hydride (NaH); mono-bromomethyl TPE affording DA-AIE-M (yield: 58%), bis-bromomethyl TPE affording DA-AIE-D (yield: 30%). The reaction intermediate was confirmed by ^1^H NMR, and the two final compounds were identified by ^1^H/^13^C NMR and high-resolution mass spectrometry (see the data in SI). As control compounds, non-conjugated compound DA and AIE-Br were used (Figure 1).

### 3.2. Spectroscopic Study

**Figure 3:**
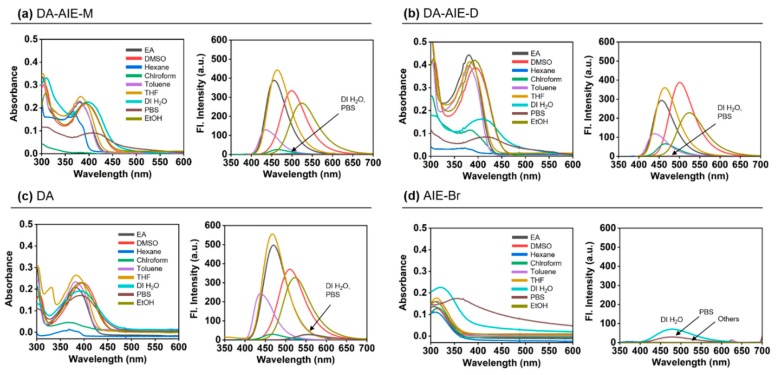
Absorption (left) and emission (right) spectra of (**a**) DA-AIE-M (10 μM), (**b**) DA-AIE-D (10 μM), (**c**) DA (10 μM), and (**d**) AIE-Br (10 μM) in various solvents at 25 °C. Solvents: EtOAc (ethyl acetate), DMSO (dimethyl sulfoxide), hexane (*n*-hexane); chloroform, toluene, THF (tetrahydrofuran), DI H_2_O (deionized water), PBS (phosphate-buffered saline, pH 7.4), and EtOH (ethanol). The emission spectra were obtained in each solvent under excitation at the maximum absorption wavelength.

The UV/vis absorption and emission spectra of synthesized DA-AIE series were measured in various solvents, and were compared with those of the control compounds, as represented in Figure 3 and Table S1 (Supporting Information).

Firstly, we observed the maximum absorption and emission wavelengths of DA-AIE series at 300–450 nm and 400–600 nm (Figure 3a,b). These optical properties were similar to a typical feature of D-A type fluorophore; (i) solvent polarity dependent wavelength shift, (ii) relatively lower emission intensity in polar solvents, (iii) negligible emission intensity in aqueous media compared with that of organic solvents, and it was correlated with control compound DA (Figure 3c). Both DA-AIE-M and DA-AIE-D showed a strong emission in aprotic solvents, including ethyl acetate (EA), toluene, tetrahydrofuran (THF), and dimethyl sulfoxide (DMSO), but negligibly weak emissions in protic polar solvents, deionized water (DI H_2_O), and phosphate-buffered saline (PBS). As we expected, the control compound AIE-Br showed emission in aqueous media (DI H_2_O, PBS), in an aggregated form, and no emissions were observed in organic solvents, in a dissolved form (Figure 3d). The emission intensity of aggregated AIE-Br in aqueous media was slightly lower than that of DA in organic solvents.

The DA-AIE series exhibited optical features that are very similar to D-A type dipolar dye, not to AIEgen. In addition, we confirmed their aggregate formation by dynamic light scattering (DLS) analysis. The DA-AIE series formed relatively uniformed aggregates, whose sizes are on a nanometer length scale (*d* = 248.9 nm and PDI = 0.102 for DA-AIE-M and *d* = 403.5 nm and PDI = 0.593 for DA-AIE-D) in DI H_2_O (Figure S1, Table S2), but they were fully dissolved in organic solvents. From the emission property monitoring with DLS analysis, we concluded that the DA-AIE series produced an aggregated form in aqueous media, but the packing of TPE moieties in the aggregates was not efficient enough to inhibit the rotation-induced non-radiative relaxation of phenyl rings. In addition, TPE emission of DA-AIE series in the aggregated form could be quenched in the excited state by closely attaching the DA moieties via a resonance energy transfer (RET) [31], followed by subsequent non-radiative decays of the DA moieties.

**Figure 4:**
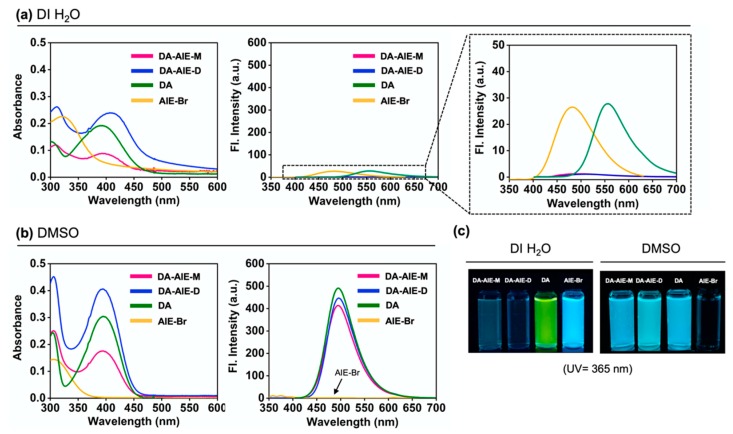
Absorption and emission spectra of DA-AIE-M (10 μM), DA-AIE-D (10 μM), DA (10 μM), and AIE-Br (10 μM) in (**a**) DI H_2_O, (**b**) DMSO at 25 °C. The emission spectra were produced in each solvent under excitation at the maximum absorption wavelength. (**c**) Photos: each compound (10 μM) is dissolved in DI H_2_O and DMSO under UV light (365 nm).

The emission properties of the compounds were summarized in two representative solvents; (i) DI H_2_O: no emission from the DA-AIE series (aggregated), emission from AIE-Br (aggregated), and emission from DA (dissolved), (ii) DMSO: emission from the DA-AIE series (dissolved, emission from DA moiety), no emission from AIE-Br (dissolved), emission from DA (dissolved) (Figure 4). Fluorescence quantum yield (QY, Φ) of the compounds in each solvent has corresponded with these results; DA-AIE-M (Φ = 0.984 in DMSO, Φ = 0.066 in DI H_2_O), DA-AIE-D (Φ = 0.678 in DMSO, Φ = 0.005 in DI H_2_O) (Table S1). The UV/vis absorption intensity of DA-AIE-D (0.26) in water was higher than that of DA-AIE-M (0.12) at 310 nm due to the two DA moieties in DA-AIE-D. A little increase in emission intensity of the DA-AIE series in DI H_2_O with increasing concentration, but their intensity is very low (Figure S2). In addition, the environment-dependent emission changes were not observed in DI H_2_O. In other words, the emission intensities of the DA-AIE series are independent of the solvent viscosity (Figure S3), metal ions, and hydrazine (N_2_H_4_), which could form hydrazone with the aldehyde functional group in DA moieties (Figure S4) [32].

### 3.3. Quantum Chemical Calculation

**Figure 5:**
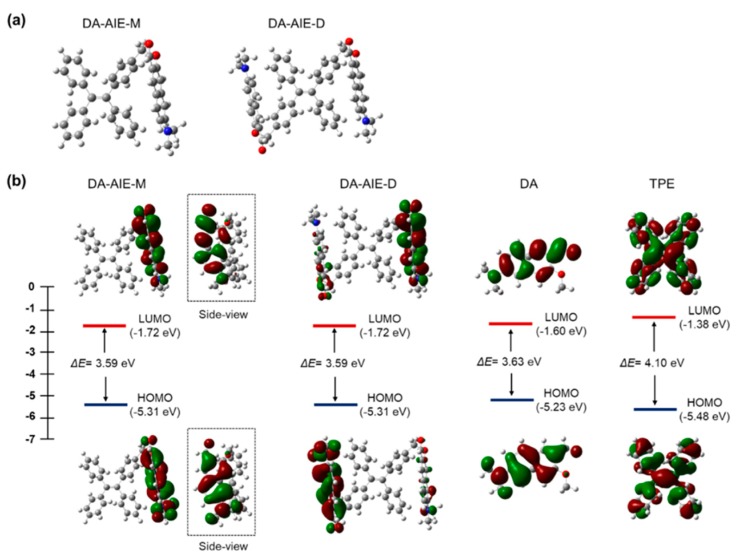
Quantum chemical calculations for DA-AIE-M, DA-AIE-D, DA, and TPE. (**a**) Optimized structures of DA-AIE-M, DA-AIE-D calculated using the DFT calculations. (**b**) The HOMO and LUMO orbitals and their energies for the compounds.

To understand the molecular conformations and optical properties of the DA-AIE series, we conducted theoretical calculations for the optimized structures and the HOMO and LUMO energies (Figure 5). In the optimized structures, the DA moieties were folded, so that the DA and TPE moieties were located close to each other. The side view images clearly showed that the DA moiety was vertically located in the plane of TPE moiety (Figure 5b). In such molecular conformations, DA moieties could disturb the packing of TPE moieties in the aggregates, and thus the AIE of TPE moieties would not be turned on in aqueous media.

The optimized molecular structures and the HOMO and LUMO of compounds were produced (Figure 5b). In DA-AIE-M and DA-AIE-D, the DA and TPE moieties are directly linked via sp^3^ carbon linker (-CH_2_-), and thus the two moieties individually contributed to the molecular orbitals (Figures S5 and S6) and the optical properties of DA-AIE-M and DA-AIE-D (Figures S7 and S8). The HOMO and LUMO of DA-AIE-M and DA-AIE-D are dictated by the DA moieties, and the absorption and emission spectra measured with DA-AIE-M and DA-AIE-D within various solvents were shown to be very similar to those of DA (Figure 3 and Figure 4). Calculated absorption and emission spectra agreed well with the experimental ones (Figures S7–S9). In DA, a relatively large electron density in the HOMO is located in the donor part (-N(Me)_2_), and in the LUMO, it is located in the acceptor part (-CHO). DA exhibits the intramolecular charge transfer (ICT) characteristics in the absorption and emission transitions. DA, DA-AIE-M, and DA-AIE-D showed almost the same bathochromic shifts in polar solvents (Figure 3).

**Figure 6:**
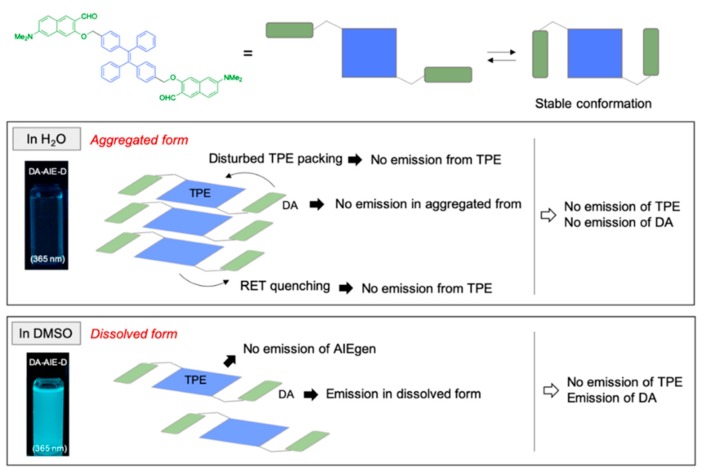
Proposed mechanism for the photophysical properties of DA-AIE-D in the aggregated form (in H_2_O) and dissolved form (in DMSO).

From the analysis of UV/vis absorption and emission spectra and quantum chemical calculation, we proposed a mechanism to explain the photophysical properties of DA-AIE-M and DA-AIE-D with a schematic illustration (Figure 6). AIE was not observed in the aggregated form of the DA-AIE series in aqueous media. That is because the DA moieties could disturb the close packing of TPE moieties, and thus allowing the phenyl groups in TPE to rotate or vibrate. Additionally, it is feasible that the RET from TPE to DA moieties occurs (Figure S10). The emission from the DA was also found to be quenched and significantly red-shifted because of the hydrogen bonding interactions in aqueous media (Figure 4a). The DA-AIE series were found to form aggregates in aqueous media but show no emission.

### 3.4. Sensing Applications in Real Water Samples

**Figure 7:**
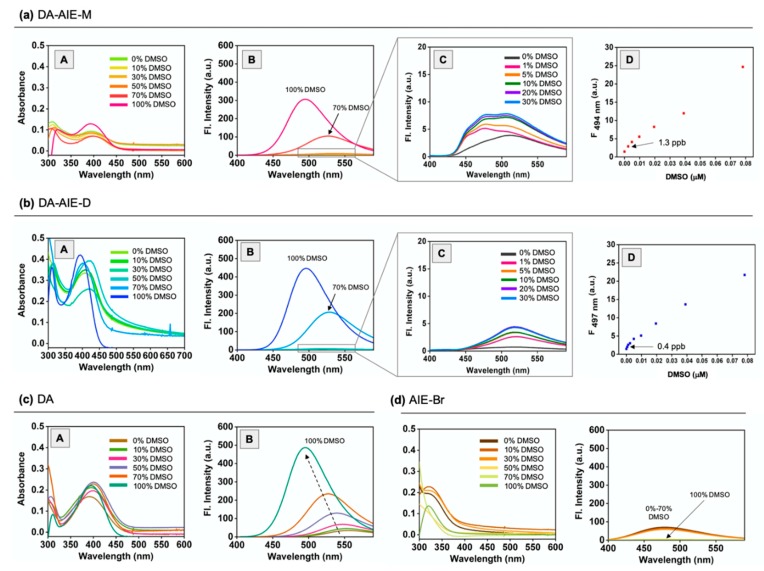
Absorbance spectra (A) and emission spectra (B) of (**a**) DA-AIE-M (10 μM), (**b**) DA-AIE-D (10 μM), (**c**) DA (10 μM), and (**d**) AIE-Br (10 μM) in DI H_2_O-DMSO mixture (0–100%). (C) Enlarged emission spectra of DA-AIE-M and DA-AIE-D in the 0–30% range of DMSO. (D) The plot of emission intensity of DA-AIE-M (at 501 nm) and DA-AIE-D (at 513 nm), in contrast to the concentration of DMSO (0–0.08 μM) in DI H_2_O, and measured at 25 °C. The emission spectra were measured under excitation at the maximum absorption wavelength. The mean value of detection limit (ppb unit) was represented and derived from triplicate measurement.

Given that the DA-AIE series exhibit a strong emission in DMSO, we have evaluated its sensing ability for the detection of residual DMSO in aqueous solution (Figure 7). First, we monitored the UV/vis absorption change and emission enhancement of the DA-AIE series in a mixture of DMSO-DI H_2_O (0–100% DMSO) (Figure 7a,b). Significant UV/vis absorption shifts were not observed in the given media. In the emission spectra, the DA-AIE series showed a strong emission and hypsochromic shift in the solutions with 70% and 100% DMSO content. The emission maximum of the DA-AIE series shifted to 527 nm for 70% DMSO and 509 nm for 100% DMSO, which is a representative feature of D-A type dipolar dye. The large emission intensity was only observed from high contents of DMSO (>70%) in aqueous media, similar to turn-on fluorescence probes, and it represented that the quenching effect of DA moiety could be diminished in a certain ratio of organic solvent. In the low content of DMSO (0–30%), a significant emission enhancement was also observed from 1% DMSO, and the enhancement factor of DA-AIE-D was more effective than DA-AIE-M, due to the completely quenched emission properties of DA-AIE-D at 0% DMSO media (Figure 7, [C]). The detection limit of the DA-AIE series was 1.3 ppb for DA-AIE-M and 0.4 ppb for DA-AIE-D, according to the signal-to-noise ratio above 3 (Figure 7, [D]). The control compound DA showed a similar property with the DA-AIE series, but the emission gradually increased depending on the DMSO ratio with hypsochromic shift (Figure 7c). As expected, the control compound AIE-Br showed emission in the mixtures with 0–70% DMSO due to the aggregate formation, and no emission was observed at 100% DMSO solution (Figure 7d). Because a similar result was observed in EtOH (Figure S11), this system could be applied for the detection of water-miscible organic solvents in aqueous media.

**Figure 8:**
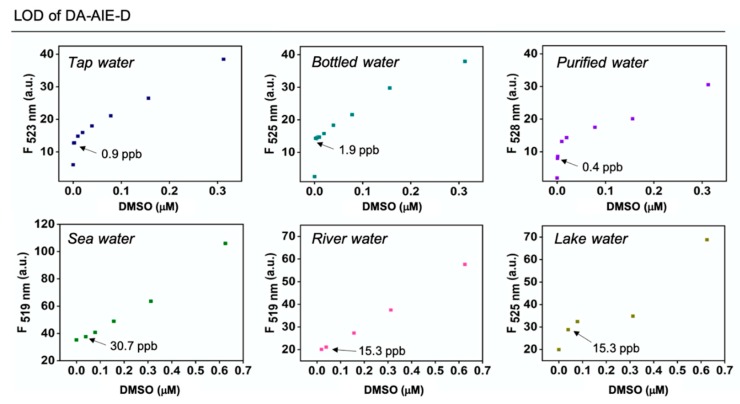
Detection limit for the DMSO in real water samples. A plot of emission intensity of DA-AIE-D (10 μM) in tap water, bottle water, purified water, sea water, river water, and lake water, in the presence of DMSO (0–0.625 μM) at 25 °C. The emission spectrum was measured under excitation at the maximum absorption wavelength. The mean value of detection limit (ppb unit) was represented and derived from triplicate measurement.

In order to further explore the potential use of the DA-AIE series for detecting a trace of DMSO, we measured the limit of detection (LOD) values for DMSO in real water samples; tap water, bottled water (commercial drinking water), purified water, sea water, river water, and lake water, by using DA-AIE-D, which was found to be more sensitive than DA-AIE-M (Figure 8). The water samples were spiked with a small amount of DMSO (0-0.625 μM) and then DA-AIE-D was added to monitor the detection limit. DA-AIE-D showed a low detection limit (<1.9 ppb) in tap water, bottled water, and purified water. In the water samples from environmental sources (sea, river, and lake), DA-AIE-D showed slightly higher LOD values for DMSO (<30.7 ppb), but the LOD values were still small enough to monitor a trace of DMSO in real water samples.

## 4. Conclusions

In conclusion, for the first time, we prepared articulated structures of D-A type dipolar dye with AIEgen. The DA-AIE series were prepared by connecting a naphthalene-based dipolar dye to a tetraphenylethylene backbone; DA-AIE-M (mono-DA-substitute), DA-AIE-D (bis-DA-substitute). We systematically analyzed the photophysical properties of the DA-AIE series with quantum chemical calculation, and demonstrated its optical properties for detecting a trace of DMSO in real water samples. We believe our current fundamental study will provide a foundation for correlated studies on DA type dipolar dyes and AIEgens, and encourage further applications in various research areas.

## Supplementary Materials

The following are available online at https://www.mdpi.com/1996-1944/13/8/1939/s1, Figure S1: Mean hydrodynamic diameter (intensity distribution) of DA-AIE-M (black line) and DA-AIE-D (red line) in DI H_2_O at 25 °C, Figure S2: Absorption and emission spectra of (a) DA-AIE-M (10–50 μM) and (b) DA-AIE-D (10–50 μM) in DI H_2_O at 25 °C. (b) Absorption and emission spectra of DA-AIE-D (10–50 μM) in DI H_2_O after 1 min at 25 °C. The emission spectra were measured under excitation at the maximum absorption wavelength, Figure S3: Absorption spectra (left) and emission spectra (right) of (a) DA-AIE-M (10 μM), (b) DA-AIE-D (10 μM), (c) DA (10 μM), and (d) AIE-Br (10 μM) in DI H_2_O-ethylene glycol (ethane-1,2-diol) mixture (0–50%) at 25 °C. The emission spectra were measured under excitation at the maximum absorption wavelength, Figure S4: Emission spectra of DA-AIE-M (10 μM) and DA-AIE-D (10 μM) after adding (a) metal ions (50 eq) and (b) hydrazine solution (0–1 mM) in DI H_2_O, measured after 1 min at 25 °C. Metal ions: AuCl_3_, FeCl_3_, FeCl_2_, CaCl_2_, CuCl_2_, HgCl_2_, AgCl_2_, CdCl_2_, NiCl_2_, NaCl_2_, PdCl_2_, KCl, L-cysteine, L-glutathione, L-lysine, DL-homocysteine. The emission spectra were measured under excitation at the maximum absorption wavelength, Figure S5: The molecular orbitals of the compounds, obtained using the DFT method (B3LYP-D3/6-31G(d)). The DA and TPE moieties individually contribute to DA-AIE-M and DA-AIE-D, Figure S6: Energy levels of the compounds, absorption transitions (solid upward arrows), and emission transitions (downward dashed arrows). The black lines are the energy levels associated with TPE, while the red lines are the energy levels associated with DA. Absorption and emission transitions in DA-AIE-M and DA-AIE-D occur locally in DA and TPE moieties, Figure S7: (a) and (b) Calculated absorption spectra of DA-AIE-M and DA-AIE-D. Dominant absorption transitions are indicated. (c) and (d) Natural transition orbitals (NTOs) associated with the corresponding absorption transitions, Figure S8: (a) and (b) Calculated emission spectra of DA-AIE-M and DA-AIE-D. Dominant emission transitions are indicated. (c) and (d) Natural transition orbitals (NTOs) associated with the corresponding emission transitions, Figure S9: (a) Absorption spectra of compounds in DMSO. (b), (c), and (d) Emission spectra of compounds measured at different excitation wavelengths, Figure S10: Absorption spectrum of DA and emission spectrum of AIE-Br (a) in DMSO and (b) in DI H_2_O. Absorption and emission spectra are significantly overlapped, and thus a resonance energy transfer from TPE to DA can potentially occur, Figure S11: Absorbance spectra (A) and emission spectra (B) of (a) DA-AIE-M (10 μM) and (b) DA-AIE-D (10 μM) in DI H_2_O-EtOH mixture (0–100%). The emission spectra were measured under excitation at the maximum absorption wavelength, Table S1: Photophysical properties of DA-AIE-M (10 μM) and DA-AIE-D (10 μM) in various solvents (EA: ethyl acetate, DMSO: dimethyl sulfoxide, hexane: n-Hexane, chloroform, toluene, THF: tetrahydrofuran, DI H_2_O: deionized water, PBS: phosphate-buffered saline, EtOH: ethanol), Table S2: The mean diameter and polydispersity index (PDI) of the aggregates of DA-AIE-M, DA-AIE-D, DA, and AIE-Br in DI H_2_O are measured using the dynamic light scattering (DLS) method. The mean and standard deviation were calculated from three data points. n.a.: not available.
