# Supplementary material for: Articulated Structures of D-A Type Dipolar Dye with AIEgen: Synthesis, Photophysical Properties, and Applications
Source: Materials (Basel). 2020 Apr 20;13(8):1939. doi: 10.3390/ma13081939 (PMC7216255; doi:10.3390/ma13081939)
Supplement: Supplementary file 1 [file materials-13-01939-s001.pdf]

materials

MDPI

SUPPLEMENTARY INFORMATION

Articulated Structures of D-A Type Dipolar Dye with AIEgen: Synthesis, Photophysical Properties, and Applications

Na Hee Kim [1,†], Byeong Wook Kim [2,†], Youngseo Kim [3,†], Junho K. Hur [1,4], Junyang Jung [1,5], Yohan Oh [6,7,*], Sungnam Park [3,*], B. Moon Kim [2,*] and Dokyoung Kim [1,5,8,9,*]

[1] Department of Biomedical Science, Graduate School, Kyung Hee University, Seoul 02447, Korea; pionaheek@gmail.com (N.H.K.); jhur@khu.ac.kr (J.K.H.); jjung@khu.ac.kr (J.J.)
[2] Department of Chemistry, College of Natural Sciences, Seoul National University, Seoul 08826, Korea; kbo0528@snu.ac.kr
[3] Department of Chemistry, Korea University, Seoul 02841, Korea; youngseo1110@hanmail.net
[4] Department of Pathology, College of Medicine, Kyung Hee University, Seoul 02447, Korea
[5] Department of Anatomy and Neurobiology, College of Medicine, Kyung Hee University, Seoul 02447, Korea
[6] Department of Biomedical Science, Graduate School of Biomedical Science and Engineering, Hanyang University, Seoul 04763, Korea
[7] Department of Biochemistry and Molecular Biology, College of Medicine, Hanyang University, Seoul 04763, Korea
[8] Center for Converging Humanities, Korea University, Seoul 02841, Korea
[9] Medical Research Center for Bioreaction to Reactive Oxygen Species and Biomedical Science Institute, School of Medicine, Graduate School, Kyung Hee University, Seoul 02841, Korea
* Correspondence: yoh@hanyang.ac.kr (Y.O.); spark8@korea.ac.kr (S.P.); kimbm@snu.ac.kr (B.M.K.); dkim@khu.ac.kr (D.K.); Tel.: +82-02-961-0297 (D.K.)
† These authors contributed equally to this work.

Received: 1 April 2020; Accepted: 17 April 2020; Published: 20 April 2020

This file includes:

Supporting Figures: Figure S1 to S11

Supporting Tables: Table S1 to S2

^{1}H and ^{13}C NMR of synthesized compounds

Supporting Figures

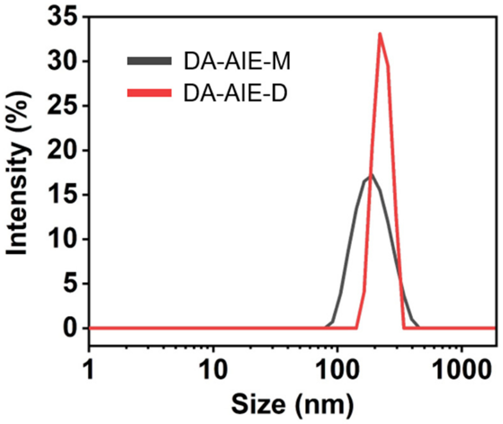

Figure S1. Mean hydrodynamic diameter (intensity distribution) of DA-AIE-M (black line) and DA-AIE-D (red line) in DI H_2O at 25 °C.

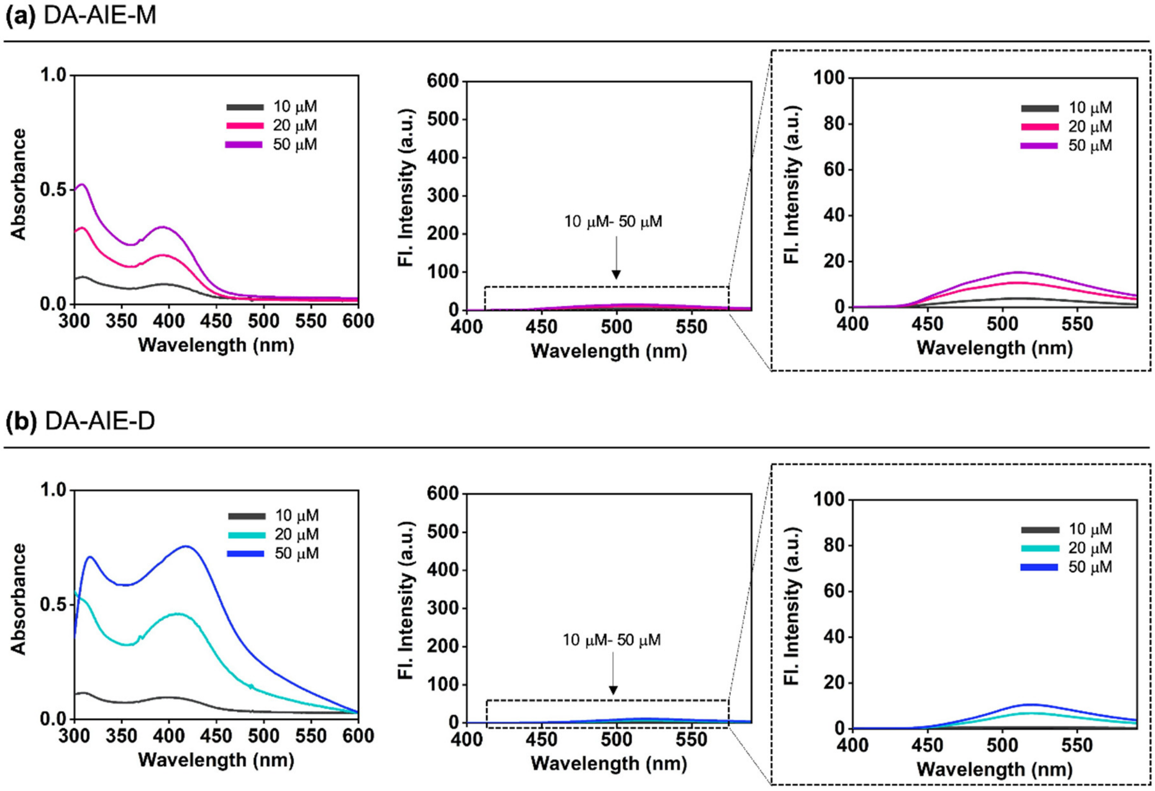

Figure S2. Absorption and emission spectra of (**a**) DA-AIE-M (10–50 μM) and (**b**) DA-AIE-D (10–50 μM) in DI H_2O at 25 °C. (**b**) Absorption and emission spectra of DA-AIE-D (10–50 μM) in DI H_2O after 1 min at 25 °C. The emission spectra were measured under excitation at the maximum absorption wavelength.

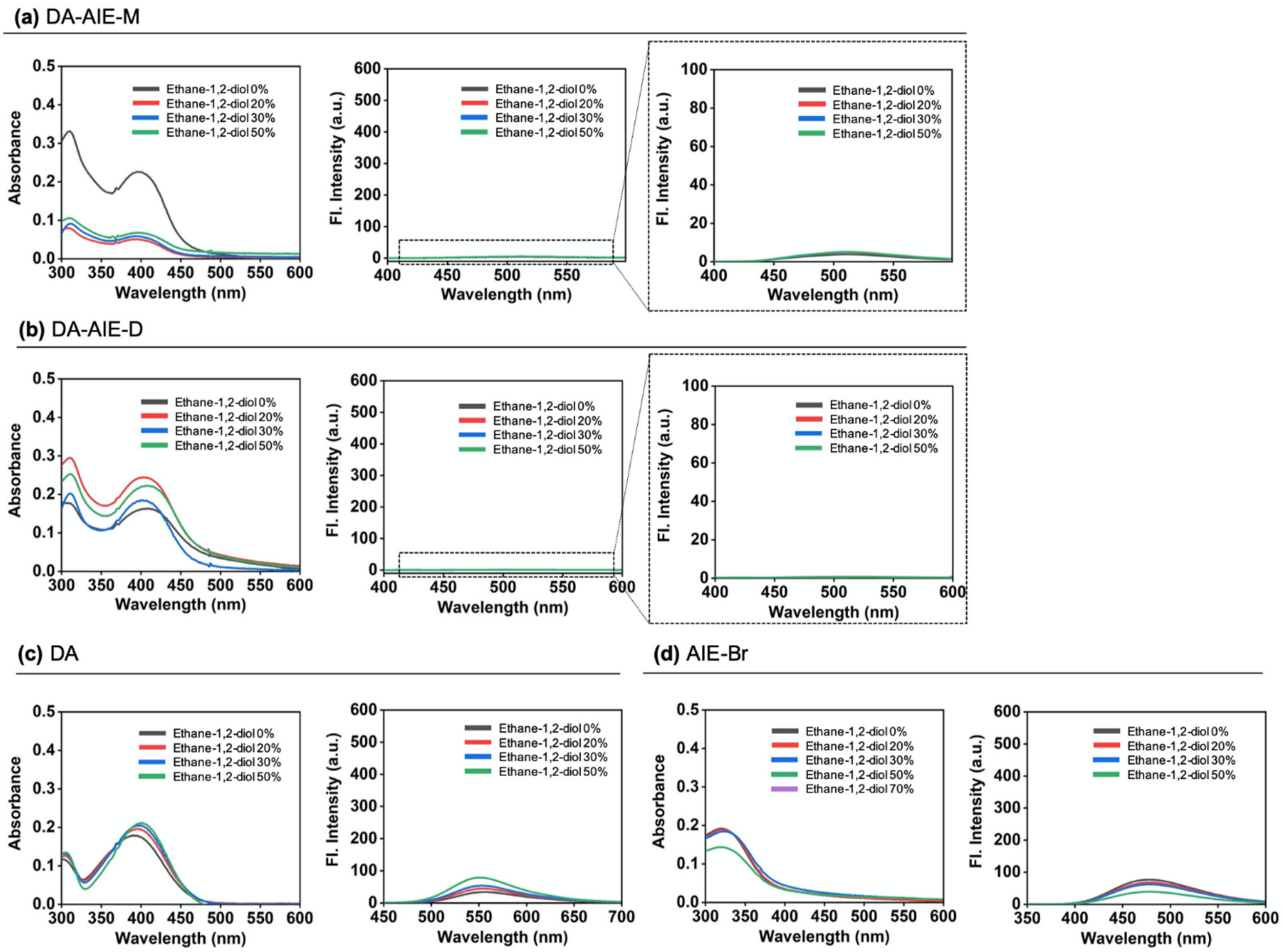

Figure S3. Absorption spectra (left) and emission spectra (right) of (**a**) DA-AIE-M (10 µM), (**b**) DA-AIE-D (10 µM), (**c**) DA (10 µM), and (**d**) AIE-Br (10 µM) in DI H_2O-ethylene glycol (ethane-1,2-diol) mixture (0–50%) at 25 °C. The emission spectra were measured under excitation at the maximum absorption wavelength.

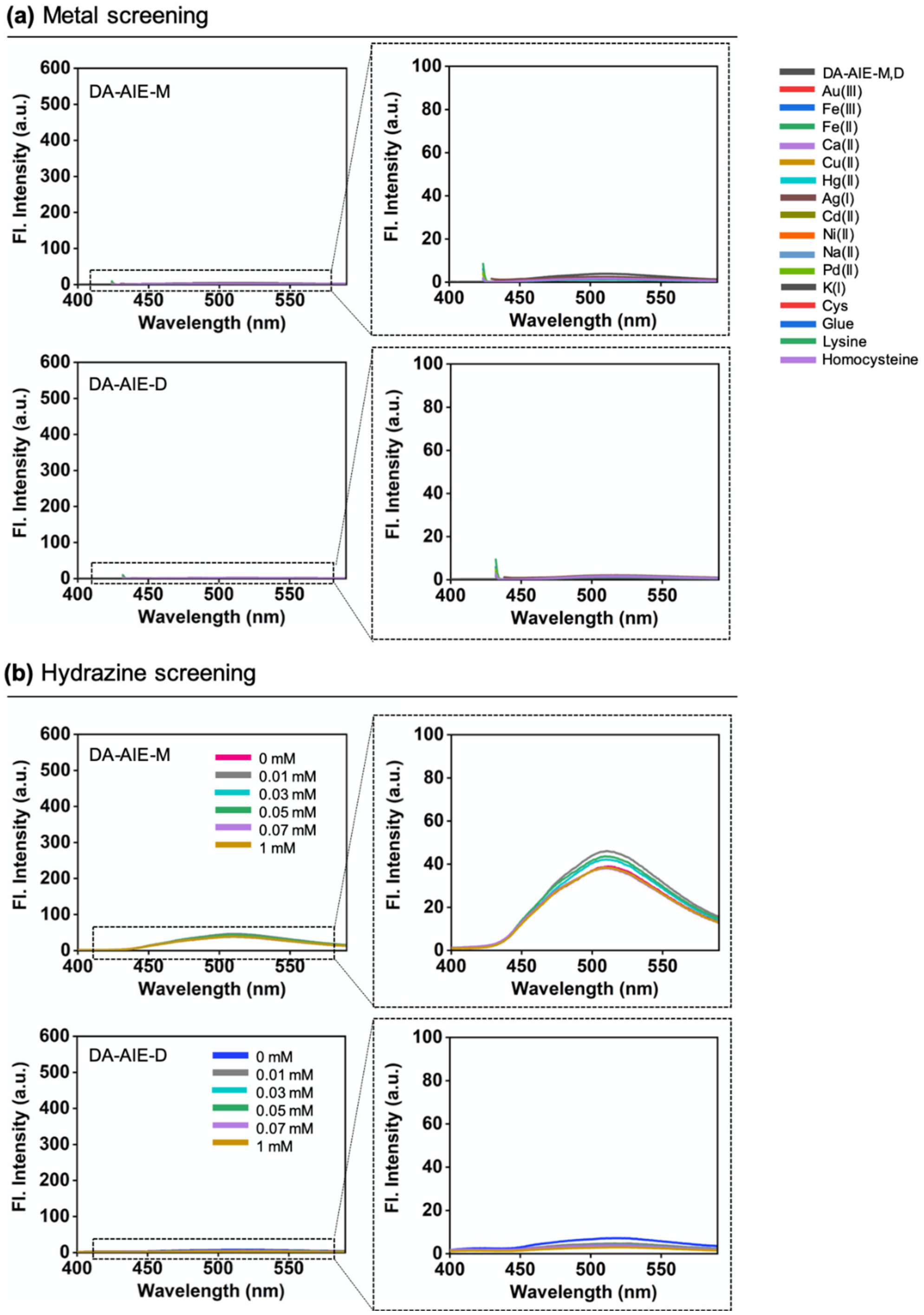

Figure S4. Emission spectra of DA-AIE-M (10 μM) and DA-AIE-D (10 μM) after adding (**a**) metal ions (50 eq) and (**b**) hydrazine solution (0–1 mM) in DI H_2O, measured after 1 min at 25 °C. Metal ions: $AuCl_3$, $FeCl_3$, $FeCl_2$, $CaCl_2$, $CuCl_2$, $HgCl_2$, $AgCl_2$, $CdCl_2$, $NiCl_2$, $NaCl_2$, $PdCl_2$, KCl, L-cysteine, L-glutathione, L-lysine, DL-homocysteine. The emission spectra were measured under excitation at the maximum absorption wavelength.

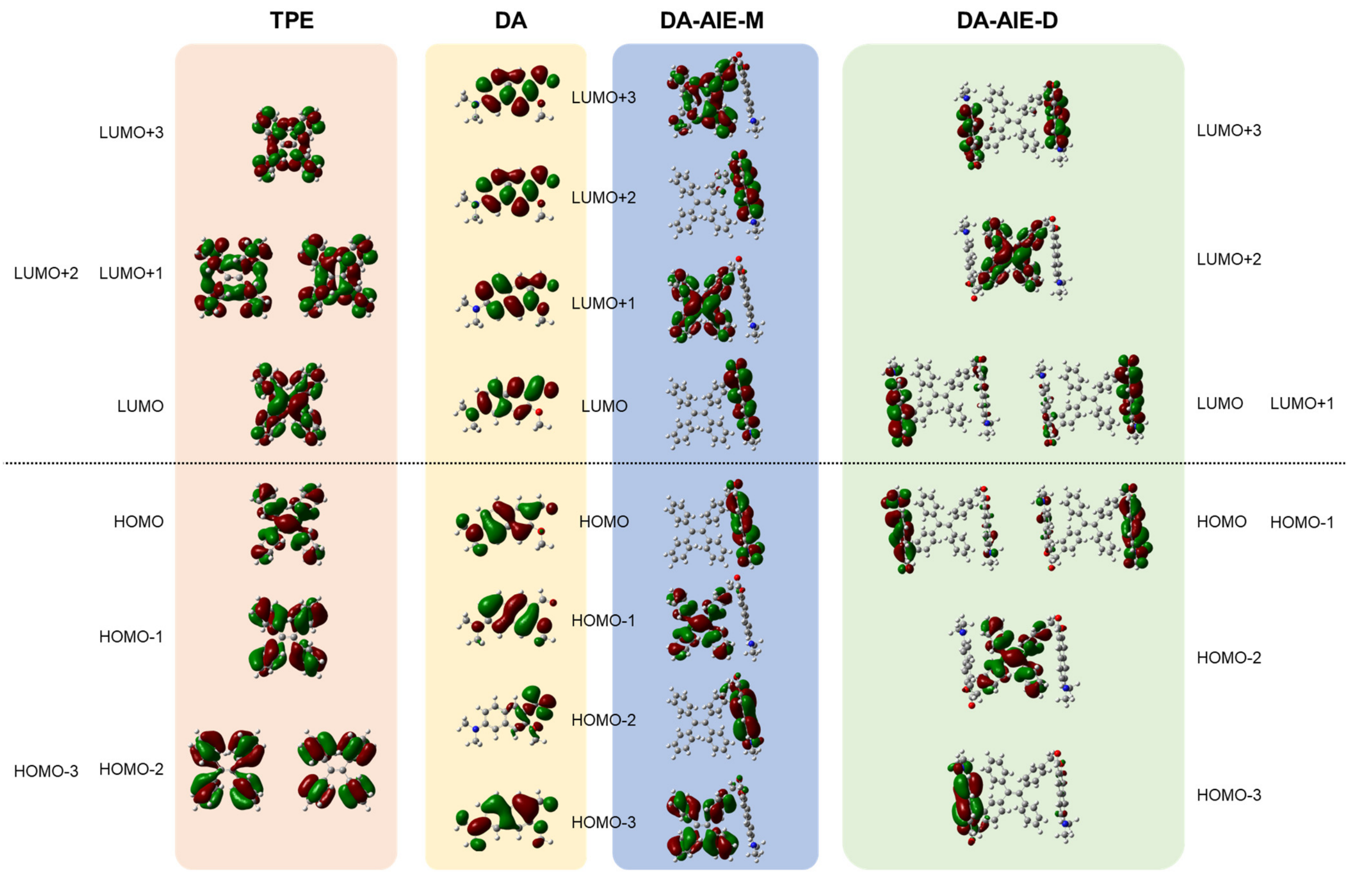

Figure S5. The molecular orbitals of the compounds, obtained using the DFT method (B3LYP-D3/6-31G(d)). The DA and TPE moieties individually contribute to DA-AIE-M and DA-AIE-D.

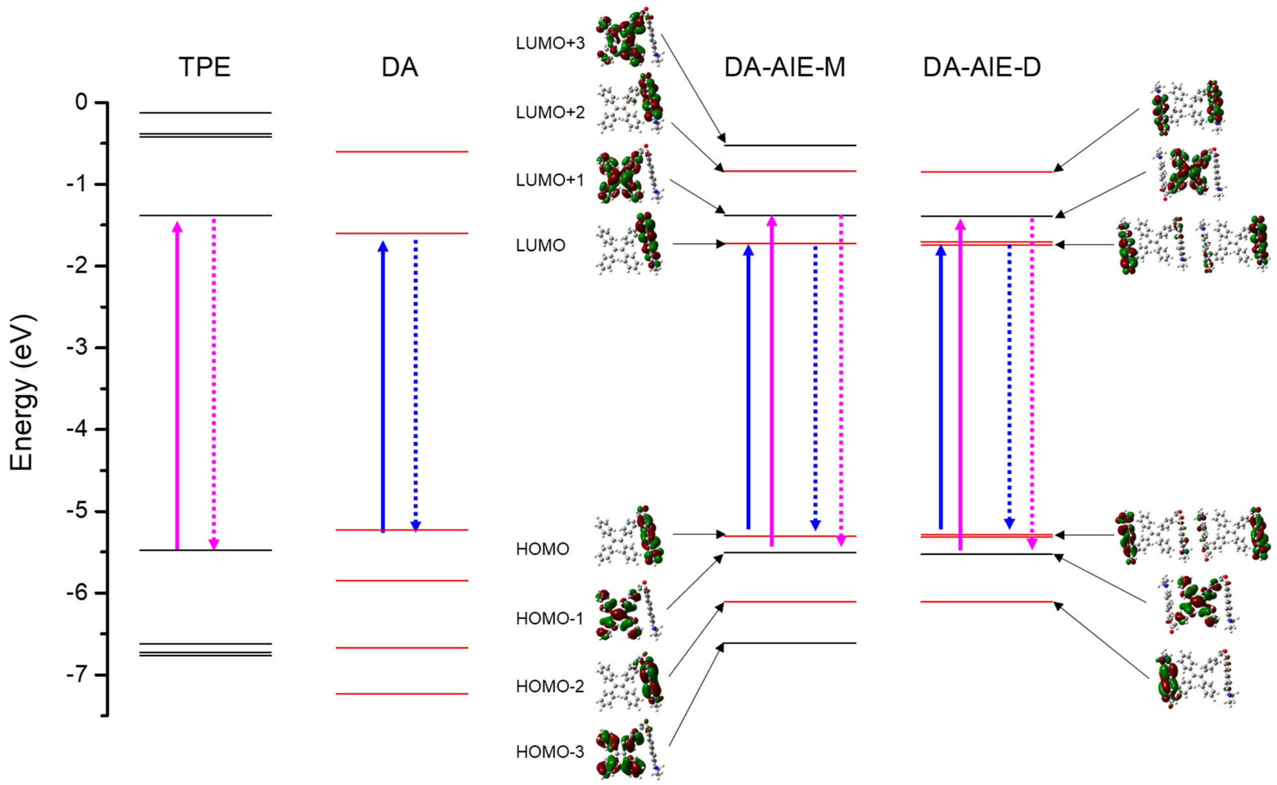

Figure S6. Energy levels of the compounds, absorption transitions (solid upward arrows), and emission transitions (downward dashed arrows). The black lines are the energy levels associated with TPE, while the red lines are the energy levels associated with DA. Absorption and emission transitions in DA-AIE-M and DA-AIE-D occur locally in DA and TPE moieties.

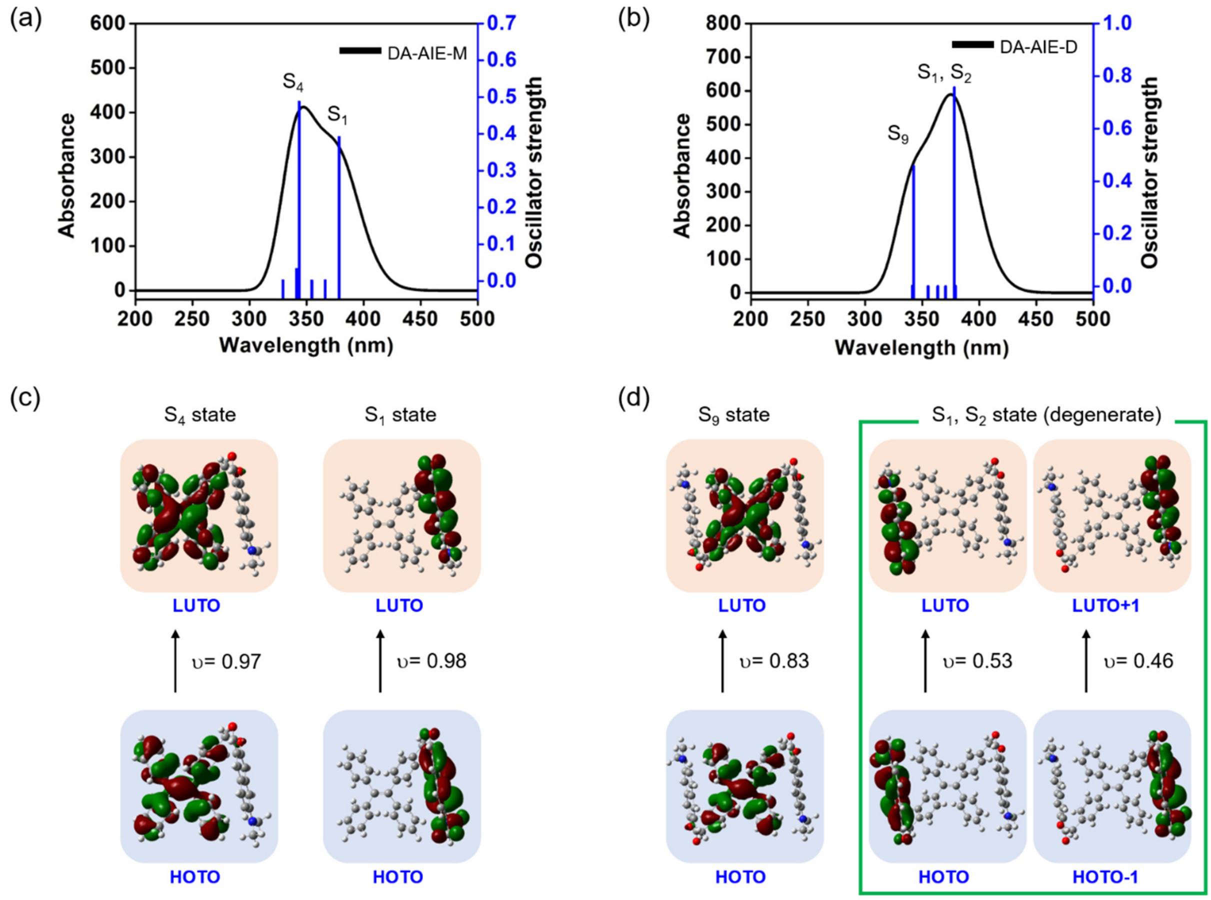

Figure S7. (**a**) and (**b**) Calculated absorption spectra of DA-AIE-M and DA-AIE-D. Dominant absorption transitions are indicated. (**c**) and (**d**) Natural transition orbitals (NTOs) associated with the corresponding absorption transitions.

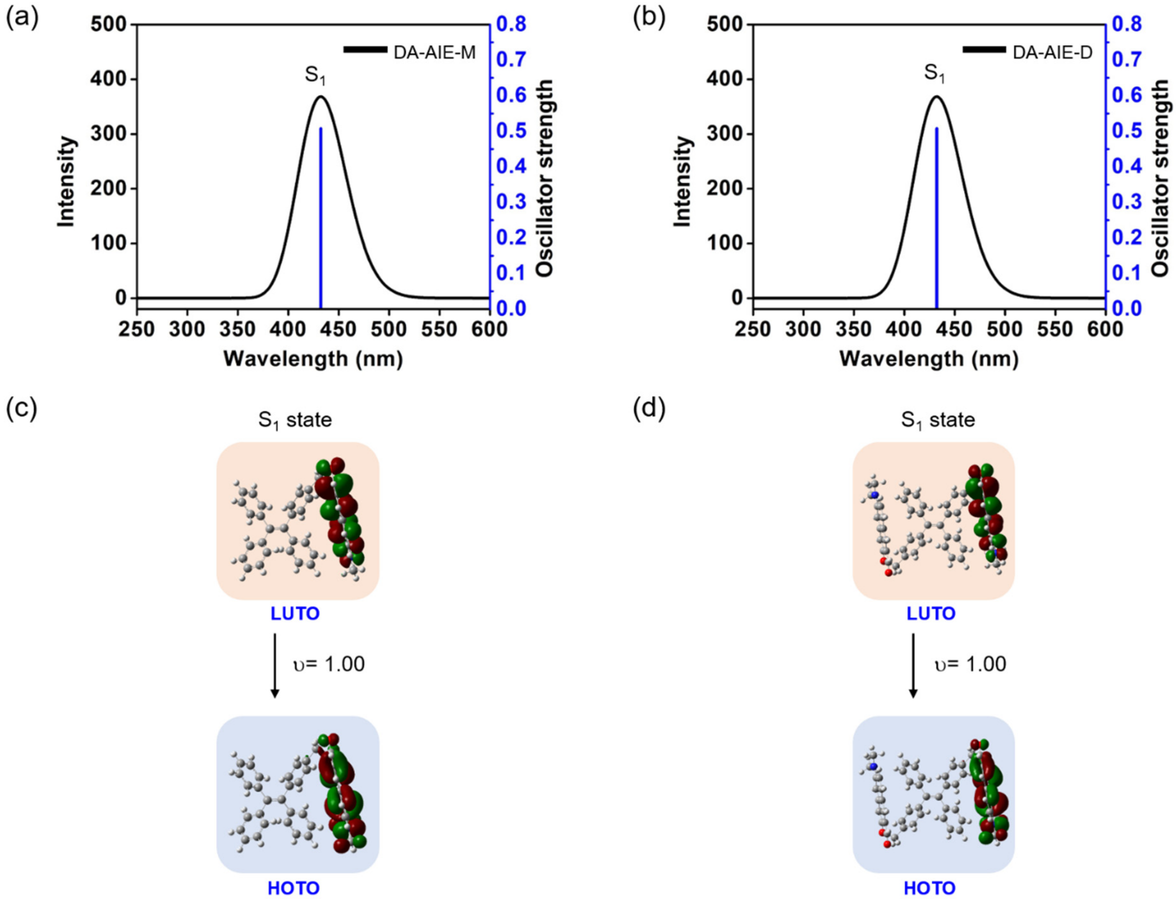

Figure S8. (**a**) and (**b**) Calculated emission spectra of DA-AIE-M and DA-AIE-D. Dominant emission transitions are indicated. (**c**) and (**d**) Natural transition orbitals (NTOs) associated with the corresponding emission transitions.

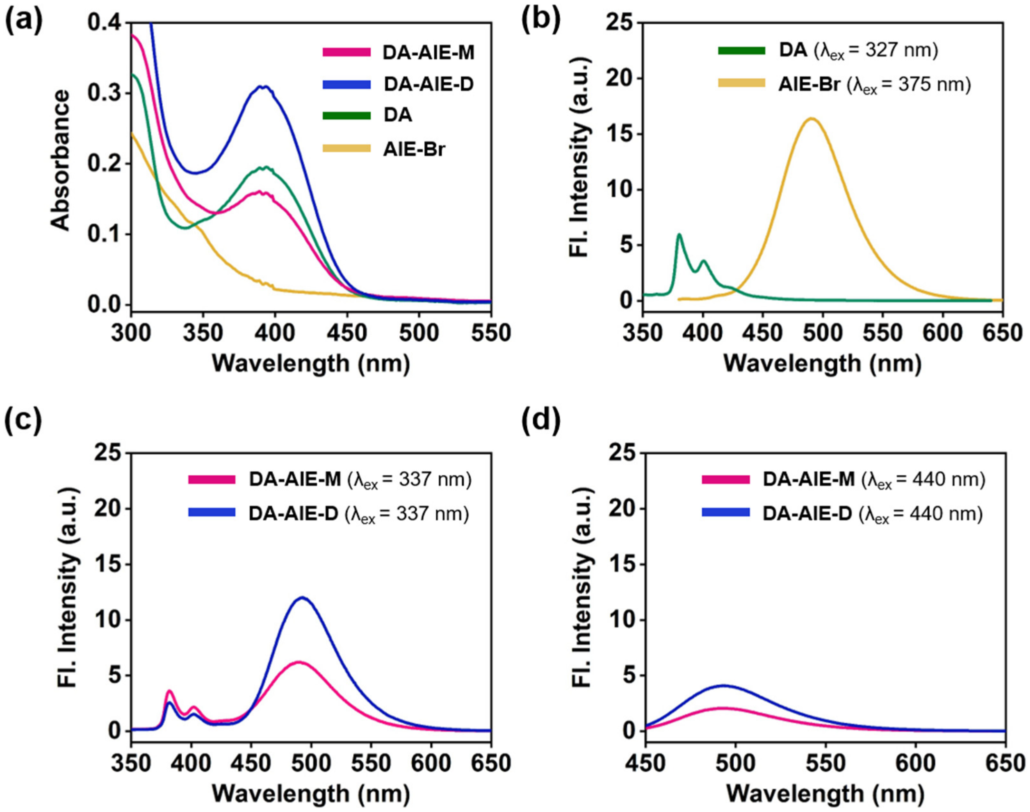

Figure S9. (**a**) Absorption spectra of compounds in DMSO. (**b**,**c**,**d**) Emission spectra of compounds measured at different excitation wavelengths.

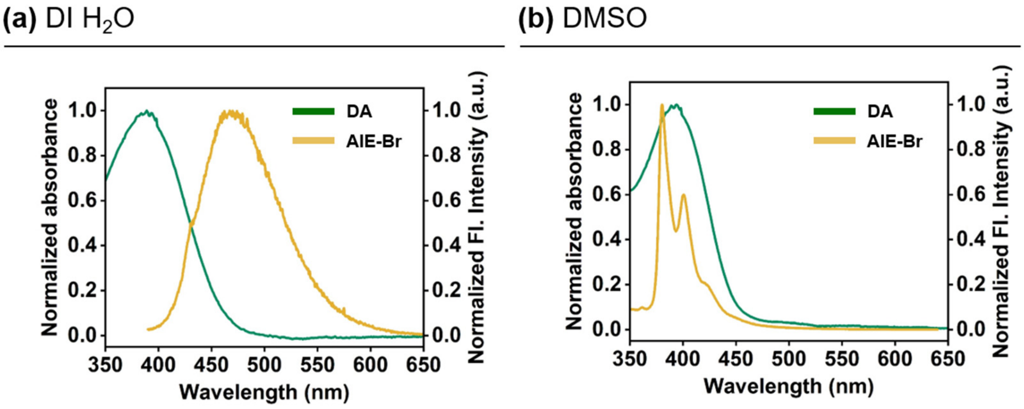

Figure S10. Absorption spectrum of DA and emission spectrum of AIE-Br (**a**) in DMSO and (**b**) in DI H_2O. Absorption and emission spectra are significantly overlapped, and thus a resonance energy transfer from TPE to DA can potentially occur.

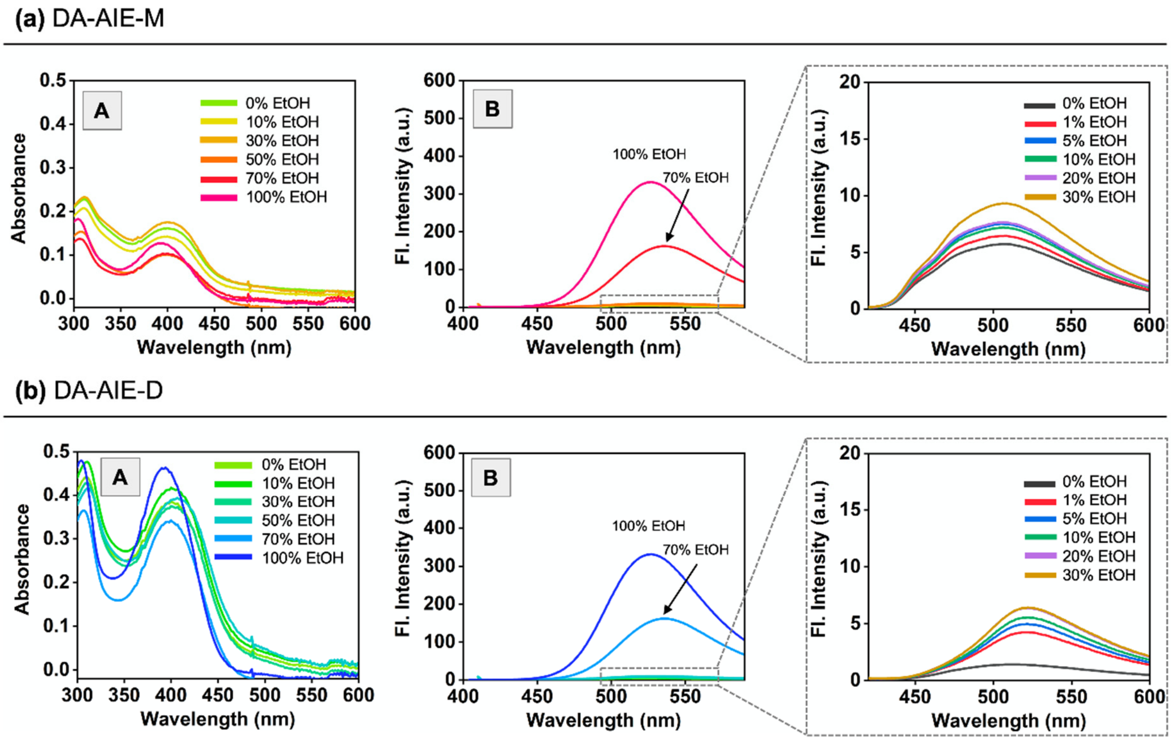

Figure S11. Absorbance spectra (A) and emission spectra (B) of (**a**) DA-AIE-M (10 µM) and (**b**) DA-AIE-D (10 µM) in DI H_2O-EtOH mixture (0–100%). The emission spectra were measured under excitation at the maximum absorption wavelength.

Supporting Tables

Table S1. Photophysical properties of DA-AIE-M (10 µM) and DA-AIE-D (10 µM) in various solvents (EA: ethyl acetate, DMSO: dimethyl sulfoxide, hexane: *n*-Hexane, chloroform, toluene, THF: tetrahydrofuran, DI H_2O: deionized water, PBS: phosphate-buffered saline, EtOH: ethanol).

Compounds	Solvents	λ_{abs} (nm)	ε (L mol^{-1} cm^{-1})	λ_{fl} (nm)	Stokes shift	Q.Y.
	EA	381	22,459	456	75	-
	DMSO	394	21,849	500	106	0.984
	Hexane	370	17,519	399	29	-
	Chloroform	369	631	464	95	-
DA-AIE-M	Toluene	380	23,067	435	55	-
	THF	382	24,928	464	82	-
	DI H_2O	396	22,593	501	105	0.066
	PBS	407	911	503	96	-
	EtOH	390	22,103	524	134	-
	EA	380	44,421	456	76	-
	DMSO	394	38,804	500	106	0.678
	Hexane	371	3,649	394	23	-
	Chloroform	383	11,495	465	82	-
DA-AIE-D	Toluene	381	39,714	438	57	-
	THF	382	41,475	464	82	-
	DI H_2O	407	16,339	513	106	0.005
	PBS	415	8,482	513	98	-
	EtOH	394	42,044	524	130	-
DA	DMSO	395	22,922	510	115	0.141
	DI H_2O	390	19,216	556	166	0.184
AIE-Br	DMSO	313	12,715	375	62	0.044
	DI H_2O	321	22,556	477	156	2.216

Table S2. The mean diameter and polydispersity index (PDI) of the aggregates of DA-AIE-M, DA-AIE-D, DA, and AIE-Br in DI H_2O are measured using the dynamic light scattering (DLS) method. The mean and standard deviation were calculated from three data points. n.a.: not available.

Compounds	DA-AIE-M	DA-AIE-D	DA	AIE-Br
Mean diameter (Polydispersity index, PDI)	248.9 ± 87.02 (0.102)	403.5 ± 32.68 (0.593)	n.a.	231.0 ± 71.55 (0.082)

^{1}H, ^{13}C NMR Spectra for the Synthesized Compounds

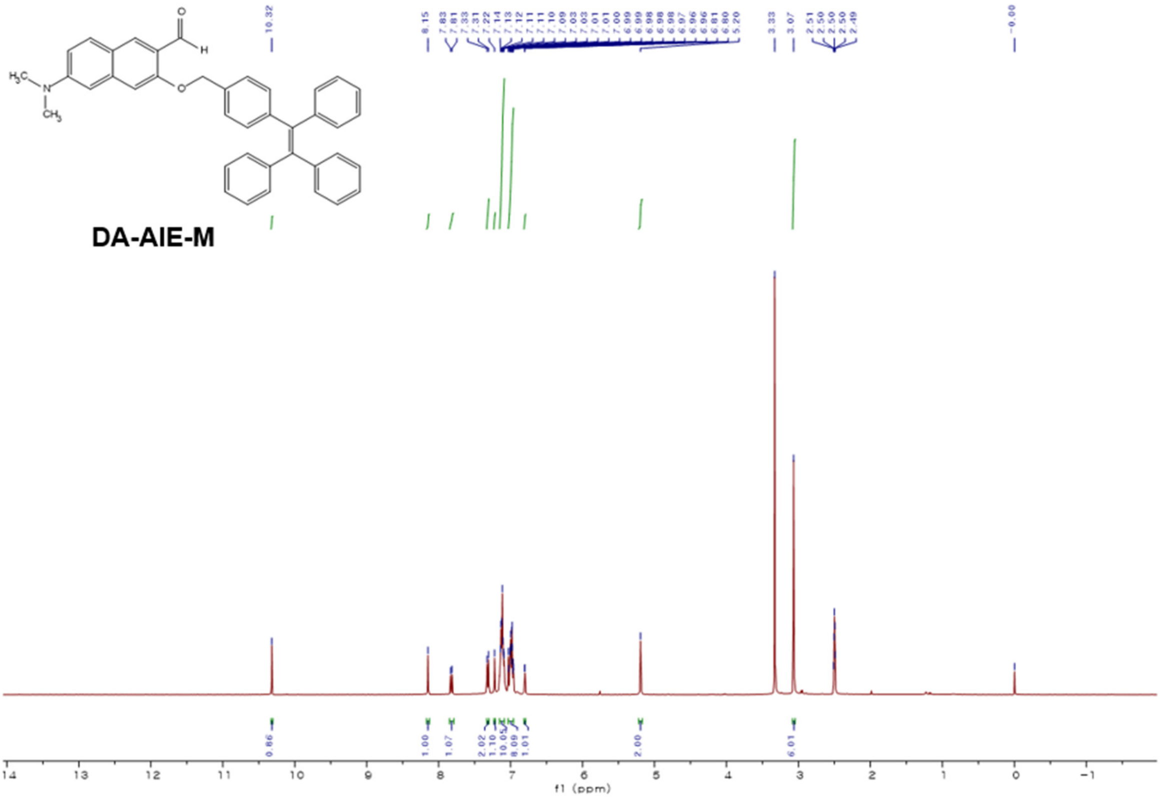

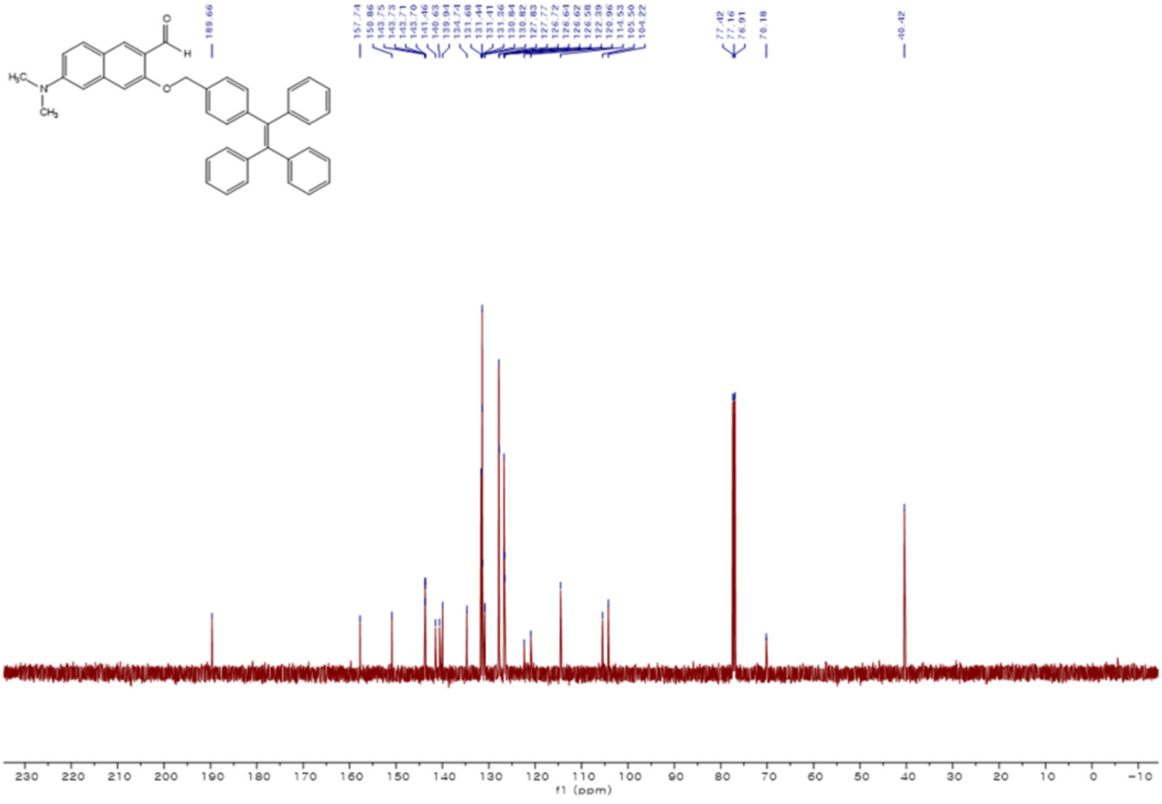

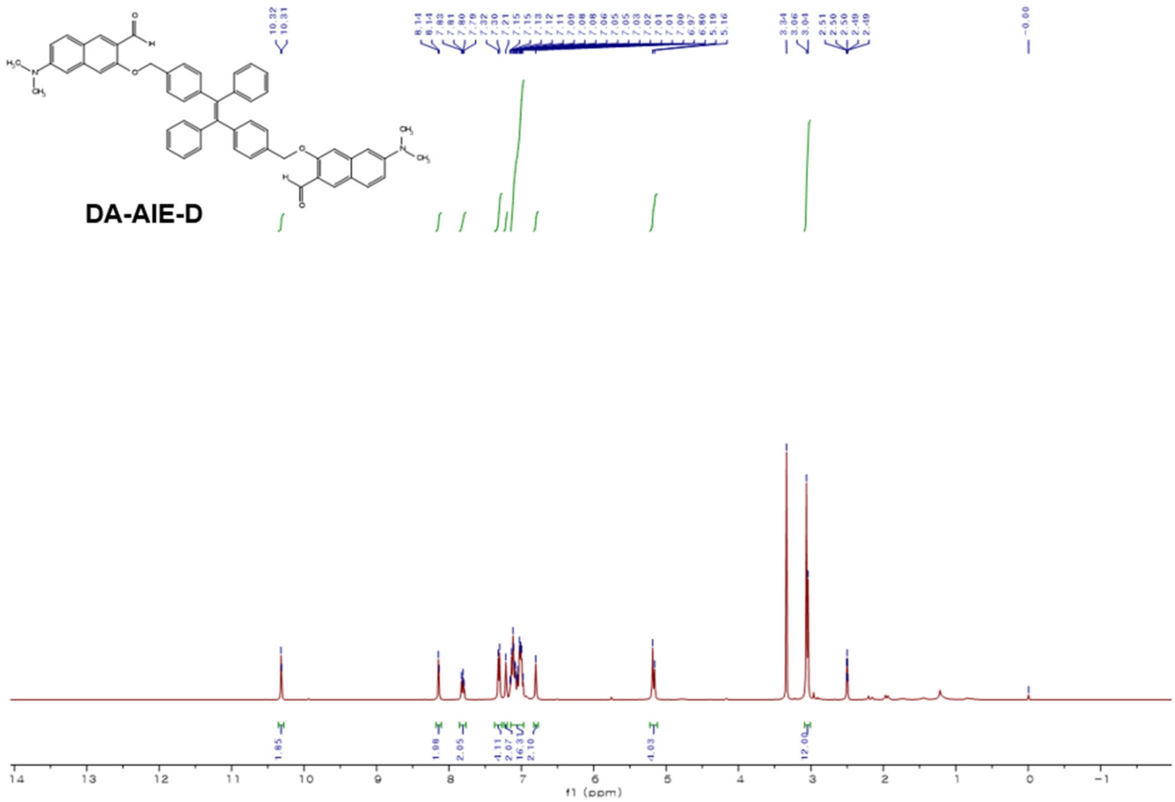

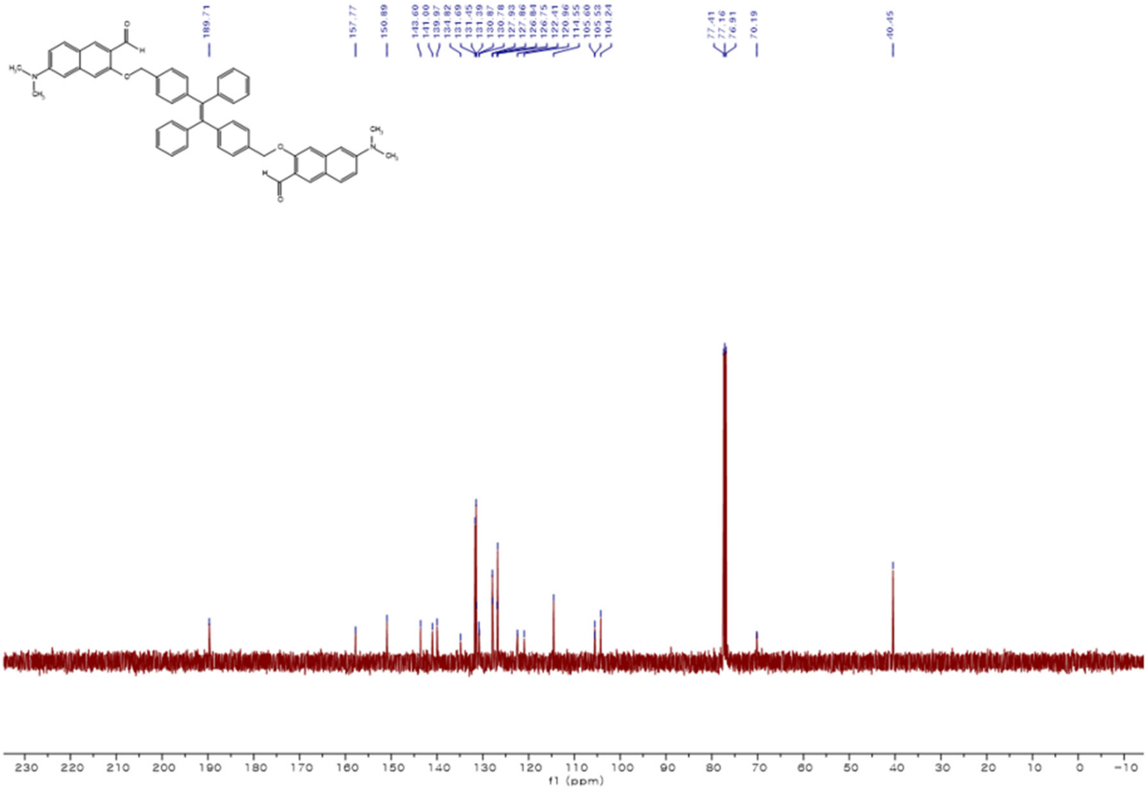

© 2020 by the authors. Submitted for possible open access publication under the terms and conditions of the Creative Commons Attribution (CC BY) license (http://creativecommons.org/licenses/by/4.0/).
